# Editorial: Plant RNA processing: discovery, mechanism and function

**DOI:** 10.3389/fpls.2024.1359415

**Published:** 2024-01-10

**Authors:** Lili Hao, Ming Chen, Dayong Li, Diego Lijavetzky

**Affiliations:** ^1^China National Center for Bioinformation, Beijing, China; ^2^National Genomics Data Center, Beijing Institute of Genomics, Chinese Academy of Sciences, Beijing, China; ^3^CAS Key Laboratory of Genome Sciences and Information, Beijing Institute of Genomics, Chinese Academy of Sciences, Beijing, China; ^4^Department of Bioinformatics, College of Life Sciences, Zhejiang University, Hangzhou, China; ^5^Beijing Vegetable Research Center (BVRC), Beijing Academy of Agriculture and Forestry Science (BAAFS), Beijing, China; ^6^Key Laboratory of Biology and Genetic Improvement of Horticultural Crops (North China), Ministry of Agriculture, Beijing, China; ^7^Beijing Key Laboratory of Vegetable Germplasm Improvement, Beijing, China; ^8^Instituto de Biología Agrícola de Mendoza (IBAM), Consejo Nacional de Investigaciones Científicas y Técnicas (CONICET), Universidad Nacional de Cuyo (FCA-UNCuyo), Mendoza, Argentina

**Keywords:** plant RNA processing, RNA editing, RNA splicing, long-noncoding RNA, microRNA

Most classes of nascent plant primary RNAs must be modified or processed in various ways to be converted to their mature forms. RNA processing is the term collectively used to describe the series of events required to produce functional RNAs. During co- and post-transcriptional regulatory stages, RNA processing such as splicing, editing, 5’ capping, 3’-end polyadenylation, nucleolytic cleavage and chemical modification, can maturate both coding RNA and non-coding RNA (ncRNAs) (e.g., long non-coding RNA, microRNA, ribosomal RNA and transfer RNA) (Marquardt et al., 2023; Sharma et al., 2023). Diverse protein factors are indispensable for RNA processing; for instance, pentatricopeptide repeat (PPR) protein can specifically bind RNA and regulate RNA editing (Small et al., 2020). Importantly, transcriptomic, bioinformatical, biochemical, and genetic studies have revealed that RNA processing affects transcriptomic and proteomic diversity, regulates gene expression, and plays essential roles in plant growth, development, biotic and abiotic stress tolerance (Zhang et al., 2018; Chaudhary et al., 2019; Sharma et al., 2023).

To present an overview of the fundamental discoveries related to the processing and modification of various classes of plant RNAs, which can generate new insights into the biological roles and underlying regulatory mechanisms of RNA processing in plant biological systems, the Research Topic of Frontiers in Plant Science on “Biological Roles of RNA Processing and its Regulations in Plants” presents 3 original research articles and 1 review report. The first article was on the roles of RNA splicing in the development of maize seed, the second one reported RNA editing’s impacts on rice drought tolerance, the third one identified lncRNAs that may be involved in rose floral scent synthesis, and the fourth one reviewed the production and regulation of miRNA in *Arabidopsis thaliana*.

Maturases regulating RNA splicing play key roles in plant development. However, the biological functions of maturases have not yet been elucidated in the important global crop maize. To this end, Fan et al. identified that *ZmnMAT1*, a gene encoding type I maturase that localizes in maize mitochondria, played a key role in splicing of *Nad1* intron 1 and *Nad4* intron 2, and further affected maize embryogenesis and endosperm development. However, no physical interactions were found between ZmnMAT1 and the splicing factors of *Nad1* intron 1 and *Nad4* intron 2, which indicated that splicing of plant group II intron was complicated. This research uncovers the function of ZmnMAT1 in mitochondrial RNA splicing and provides new insights into maize seed development.

Abiotic stress such as drought is an important factor affecting grain yield. RNA editing mainly modulated by PPR proteins in mitochondria and/or plastids is related to rice drought tolerance. To elucidate the mechanism of RNA editing in response to drought in rice, Luo et al. systematically analyzed mitochondrial RNA editing in both upland and lowland rice. They found that editing levels of numerous RNA editing sites were affected by drought and ecotype and editing efficiency presents negative correlation with drought tolerance. Furthermore, they found that mutations in two PPR genes, *ppr035* and *ppr406* improved drought tolerance and upland rice containing special haplotypes of these two PPR genes were more tolerant of drought. Overall, they elucidated the mechanism of rice drought tolerance from the perspective of RNA editing.

Long non-coding RNAs (lncRNAs) are essential for RNA processing and diverse biological processes. However, investigation on lncRNAs and their functions in rose, the important economical plant, is deficient. By analyzing RNA sequencing (RNA-seq) data from the scented variety ‘Tianmidemeng’ (*Rosa hybrida*), Shi et al. identified numerous well-annotated and novel lncRNAs that probably regulate synthase genes and transcription factors related to floral scent synthesis. Furthermore, they verified the biological function of a lncRNA TCONS_00008447 on floral scent synthesis. Collectively, they provided insights into the roles of lncRNA on rose floral scent synthesis.

Although the molecular framework of plant miRNA biogenesis and function are relatively clear, the linkage between different regulation steps are not fully understood. Accordingly, Ding and Zhang outlined the biogenesis and metabolism pathway of miRNAs in model plant *Arabidopsis thaliana*, and pointed out the limitations of studies on miRNA, which provides future directions for studies on miRNAs.

In summary, the articles in this Research Topic provide insights into mechanisms and functions of RNA splicing, RNA editing, lncRNA and miRNA in various biological processes. Fundamental researches on plant RNA processing have great prospective in the improvement of plant breeding and yields.

## Author contributions

LH: Writing – original draft, Writing – review & editing. MC: Writing – review & editing. DL: Writing – review & editing. DCL: Writing – review & editing.
